# Candidate plasma biomarkers for predicting ascending aortic aneurysm in bicuspid aortic valve disease

**DOI:** 10.1186/s13019-018-0762-1

**Published:** 2018-06-22

**Authors:** Oliver J. Harrison, Felino Cagampang, Sunil K. Ohri, Christopher Torrens, Kareem Salhiyyah, Amit Modi, Narain Moorjani, Anthony D. Whetton, Paul A. Townsend

**Affiliations:** 1 Institute of Developmental Sciences, Faculty of Medicine, University of Southampton, Southampton, UK; 2 Department of Cardiac Surgery, University Hospital Southampton, Southampton General Hospital, Tremona Road, D-level, North Wing (MP 46), Southampton, UK; 3 Sussex Cardiac Centre, Brighton, UK; 4 Department of Cardiac Surgery, Papworth Hospital NHS Foundation Trust, University of Cambridge, Cambridge, UK; 5 Stoller Biomarker Discovery Centre, Manchester Academic Health Science Centre, University of Manchester, Manchester, UK; 6 Division of Cancer Sciences, Faculty of Biology, Medicine and Health, Manchester Cancer Research Centre, Manchester Academic Health Science Centre, University of Manchester, Manchester, UK

**Keywords:** Bicuspid aortic valve, Ascending aortic aneurysm, Plasma biomarker, Risk prediction

## Abstract

**Background:**

Bicuspid aortic valve (BAV) disease is the most common congenital cardiac abnormality affecting 1–2% of the population and is associated with a significantly increased risk of ascending aortic aneurysm. However, predicting which patients will develop aneurysms remains a challenge. This pilot study aimed to identify candidate plasma biomarkers for monitoring ascending aortic diameter and predicting risk of future aneurysm in BAV patients.

**Methods:**

Plasma samples were collected pre-operatively from BAV patients undergoing aortic valve surgery. Maximum ascending aortic diameter was measured on pre-operative transoesophageal echocardiography. Maximum diameter ≥ 45 mm was classified as aneurysmal. Sequential Window Acquisition of all THeoretical Mass Spectra (SWATH-MS), an advanced mass spectrometry technique, was used to identify and quantify all proteins within the samples. Protein abundance and aortic diameter were correlated using logistic regression. Levene’s test was used to identify proteins demonstrating low abundance variability in the aneurysmal patients (consistent expression in disease), and high variability in the non-aneurysmal patients (differential expression between ‘at risk’ and not ‘at risk’ patients).

**Results:**

Fifteen plasma samples were collected (seven non-aneurysmal and 8 aneurysmal BAV patients). The mean age of the patients was 55.5 years and the majority were female (10/15, 67%). Four proteins (haemoglobin subunits alpha, beta and delta and mannan-binding lectin serine protease) correlated significantly with maximal ascending aortic diameter (*p* < 0.05, *r* = 0.5–0.6). Five plasma proteins demonstrated significantly lower variability in the aneurysmal group and may indicate increased risk of aneurysm in non-aneurysmal patients (DNA-dependent protein kinase catalytic subunit, lumican, tetranectin, gelsolin and cartilage acidic protein 1). A further 7 proteins were identified only in the aneurysmal group (matrin-3, glucose-6-phosphate isomerase, coactosin-like protein, peptidyl-prolyl cis-trans isomerase A, golgin subfamily B member 1, myeloperoxidase and 2′-deoxynucleoside 5′-phosphate N-hydrolase 1).

**Conclusions:**

This study is the first to identify candidate plasma biomarkers for predicting aortic diameter and risk of future aneurysm in BAV patients. It provides valuable pilot data and proof of principle that could be used to design a large-scale prospective investigation. Ultimately, a more affordable ‘off-the-shelf’ follow-on blood assay could then be developed in place of SWATH-MS, for use in the healthcare setting.

**Electronic supplementary material:**

The online version of this article (10.1186/s13019-018-0762-1) contains supplementary material, which is available to authorized users.

## Background

Bicuspid aortic valve (BAV) disease is the most common congenital cardiac malformation affecting 1–2% of the population [1, 2]. BAV occurs when the aortic valve forms with just two leaflets (or cusps), rather than the normal three (tricuspid aortic valve; TAV). Although between 30 and 80% of patients with BAV disease will develop clinically significant enlargement of the ascending aorta, predictors of aortic dilation are lacking [3, 4]. The consequences of undiagnosed or rapidly progressive aneurysm can be severe, with rupture and dissection frequently being fatal. There is ongoing controversy regarding optimal timing of ascending aortic surgery for patients with BAV disease [5, 6]. Current guidelines are limited to measurement of ascending aortic diameter and the extent of aortic valve disease when planning surgery for aortic aneurysm in BAV patients [7]. An ability to predict which BAV patients will develop ascending aortic aneurysm would allow better individualisation of treatment and help prevent the potentially catastrophic complications of rupture and dissection.

A biomarker may be defined as a measurable gene, protein, cell or metabolic by-product that represents a process in a defined biological system or disease state [8, 9]. One or a combination of biomarkers may be measured to give information about risk, presence and progress of a particular disease. Several studies have attempted to identify biomarkers in BAV aortopathy, however few have investigated blood biomarkers. Matrix metalloproteinases (MMPs) are implicated in the pathogenesis of BAV aortopathy, and the expression of MMP-2, MMP-8 and MMP-9 has been demonstrated to correlate with ascending aortic diameter [10–14]. Similarly, asymmetric dimethylarginine (ADMA; a nitric oxide synthase inhibitor), alpha-1-antitrypsin (α1AT; an abundant protease inhibitor) and more recently circulating endothelial microparticles (EMP), have all been shown to differ in expression between non-aneurysmal and aneurysmal BAV patients [12, 13, 15]. Currently however, measurement of the ascending aortic diameter is the only method widely used to monitor aortopathy in BAV patients [16].

Sequential Window Acquisition of all THeoretical Mass Spectra (SWATH-MS) is one of the most recent advances in protein quantification technology, combining the advantages of the shotgun (high throughput) and selected reaction monitoring (high reproducibility and consistency) mass spectrometry techniques [17]. In essence, it allows a complete recording of all fragment ions of the detectable peptide precursors present in a biological sample, and thus is ideally suited to biomarker discovery. However, to the best of our knowledge, the technique has never before been used for the purpose of biomarker discovery in BAV-associated aneurysms.

Discovery of novel biomarkers for detection and monitoring of aneurysms in BAV disease has a number of potential benefits. Firstly, a suitable biomarker could be monitored as an indicator of aortic diameter in preference to repeated imaging, particularly computerised tomography, which carries a radiation risk. Secondly, biomarkers that predict risk of future aneurysm in BAV patients would help prevent catastrophic complications and allow individualisation when planning aortic surgery. The question asked recently by Sievers et al. is extremely important; in the young patient requiring aortic valve replacement for severe aortic stenosis, with an ascending aortic diameter at the higher end of normal; should it be replaced? [18] There is a pressing need for biomarkers to facilitate a more patient-centred approach to managing BAV aortopathy. We hypothesise that a subpopulation of BAV patients without aortic aneurysms exhibit a biomarker profile that indicates they will develop ascending aortic aneurysm in future. Therefore, the two aims of this study were to correlate plasma protein concentrations with ascending aortic diameter to identify biomarkers which could be used to estimate ascending aortic diameter in BAV patients. Secondly, to identify biomarkers which are homogenously expressed in the aneurysmal BAV group but differentially expressed in non-aneurysmal BAV patients, and may signify increased risk of future aneurysm. Here we show that a number of candidate predictors for aortic diameter were identified and these may help stratify the future risk of aneurysm in BAV patients. Our study, albeit small, provides proof of principle data that offers confidence in the potential design of a larger, prospective investigation of candidate BAV biomarkers.

## Methods

### Ethical approval and sample collection

This study complied with the declaration of Helsinki. Ethical approval was granted by Hampshire B NRES committee south central (REC Ref: 11/SC/0258). The local sponsor was University Hospital Southampton R&D Department (Protocol Ref: RHM CAR 0392). Informed consent was obtained from patients admitted electively to the University Hospital Southampton, UK for aortic valve replacement and/or ascending aortic replacement. Patients with mitral valve disease (greater than mild), atherosclerosis of the ascending aorta (either on pre-operative imaging or observed intraoperatively), infective endocarditis, known genetic conditions (such as Marfan Syndrome), and those aged under 18 or over 80 were excluded from the study. Demographic information, past medical history, echocardiography data and blood results were collected. In the anaesthetic room, all patients underwent transoesophageal echocardiography (TOE) as part of the routine theatre practice. From these images, ascending aortic measurements were obtained. Aneurysm of the ascending aorta was defined as diameter ≥ 45 mm. Aortic valve morphology was defined intraoperatively by the surgeon according to the Sievers classification [19]. Fifteen plasma samples were collected (seven from non-aneurysmal and 8 from aneurysmal BAV patients). Shortly after the induction of anaesthesia 4 mL of whole blood was taken from the central venous cannula immediately after insertion. The blood was collected following our standard operating procedure (SOP) in an EDTA containing tube and immediately placed on ice before being centrifuged at 1500 rpm for 10 min at 4^o^ C. The plasma layer was aliquoted into microtubes and stored at -80^o^ C until use.

Plasma samples were subject to SWATH-MS according to the standard operating procedures of the Stoller Biomarker Discovery Centre, Manchester, UK (see Additional file 1). In brief, major plasma proteins were removed by immunodepletion following which protein concentration was measured in each sample. Samples were then digested and dried overnight before processing in a SCIEX TripleTOF 6600 variable window mass spectrometer with a 120 min run time. The presence and abundance of plasma proteins was quantified using protein spectral libraries and SWATH-MS maps.

### Statistical analysis

Statistical analysis was performed using IBM SPSS Statistics (Version 22.0). Linear regression analysis was performed to detect significant correlations between maximum aortic diameter and plasma protein levels. Levene’s test was used to identify plasma proteins demonstrating significant differences in equality of variance between non-aneurysmal and aneurysmal groups. Levene’s test assesses the probability that a difference in the variability between two populations has occurred by chance. Where Levene’s test was significantly different, scatter plots were generated and proteins displaying low variability in the aneurysmal group (and high variability in the non-aneurysmal group) were identified as potential aortopathy risk predictors. An ‘at risk’ cut-off point for candidate biomarker expression in the non-aneurysmal patients was established at 2 standard deviations above or below the mean expression in the aneurysmal group. Where candidate markers were expressed in non-aneurysmal patients at such a level, these patients were deemed hypothetically ‘at risk’. *p* < 0.05 was taken as statistically significant.

## Results

Fifteen plasma samples were collected (seven non-aneurysmal and eight aneurysmal BAV patients). The mean age of the patients was 55.5 years and the majority of patients were female (10/15; 67%, Table 1). The groups were equally matched, except none of the aneurysmal patients were prescribed statins and their pre-operative haemoglobin values were higher. Overall 826 proteins were identified across the 15 samples.

**Table 1:** Patient demographics

Demographic	Non-aneurysmal (*n* = 7)	Aneurysmal (*n* = 8)	*p*-value
Mean age (years)	59.3 ± 13.3	51.8 ± 10.4	0.24
Gender (n)			0.32
Male	3	2	
Female	4	6	
Mean body mass index (kg/m^2^)	29.9 ± 5.0	29.7 ± 5.1	0.92
Mean maximum ascending aortic diameter (mm)	40.6 ± 2.6	53.4 ± 5	**< 0.001**
Mean blood pressure (mmHg)			
Systolic	137 ± 15.6	133 ± 16.3	0.61
Diastolic	73 ± 10.4	76 ± 11.1	0.57
Smoking status (n)			0.35
Non-smoker	5	3	
Ex-smoker	2	4	
Current smoker	0	1	
Type 2 diabetes (n)	2	1	0.57
Medications (n patients taking)			
ACE inhibitor	2	1	0.57
β-blocker	3	2	0.61
Angiotensin Receptor Blocker	1	0	0.47
Calcium Channel Blocker	1	0	0.47
Statin	4	0	**0.03**
Aortic valve disease (n)			0.33
Normal	**0**	**0**	
Stenosis	**3**	**1**	
Mild	0	0	
Mod	0	0	
Severe	5	6	
Regurgitation	**2**	**2**	
Mild	1	3	
Mod	1	1	
Severe	2	3	
Mixed	**2**	**5**	
Left ventricular ejection fraction (n)			0.47
45–70%	7	6	
35–44%	0	0	
< 35%	0	0	
Unknown	0	2	
Mean preoperative blood results			
Haemoglobin (g/dl)	138 ± 15	155 ± 15	**0.047**
White cell count (× 10^9^/l)	6.9 ± 1.8	8.7 ± 2.9	0.17
Platelet count (×10^9^/l)	226 ± 38	235 ± 38	0.65
Urea (mmol/l)	5.1 ± 1.6	4.7 ± 1.1	0.58
Creatinine (μmol/l)	85 ± 28	76 ± 23	0.50
C-reactive protein (CRP; mg/l)	4.0 ± 2.2	4.9 ± 4.1	0.71
Serum cholesterol (mmol/L)	5.4 ± 0.9	5.3 ± 0.5	0.84

Mean values are given ± standard deviation. *P*-values are given for independent samples t-tests between the study groups. Significant p-values (*p* < 0.05) are highlighted in bold

### Plasma biomarkers as predictors of aortic diameter in BAV patients

**Fig. 1:**
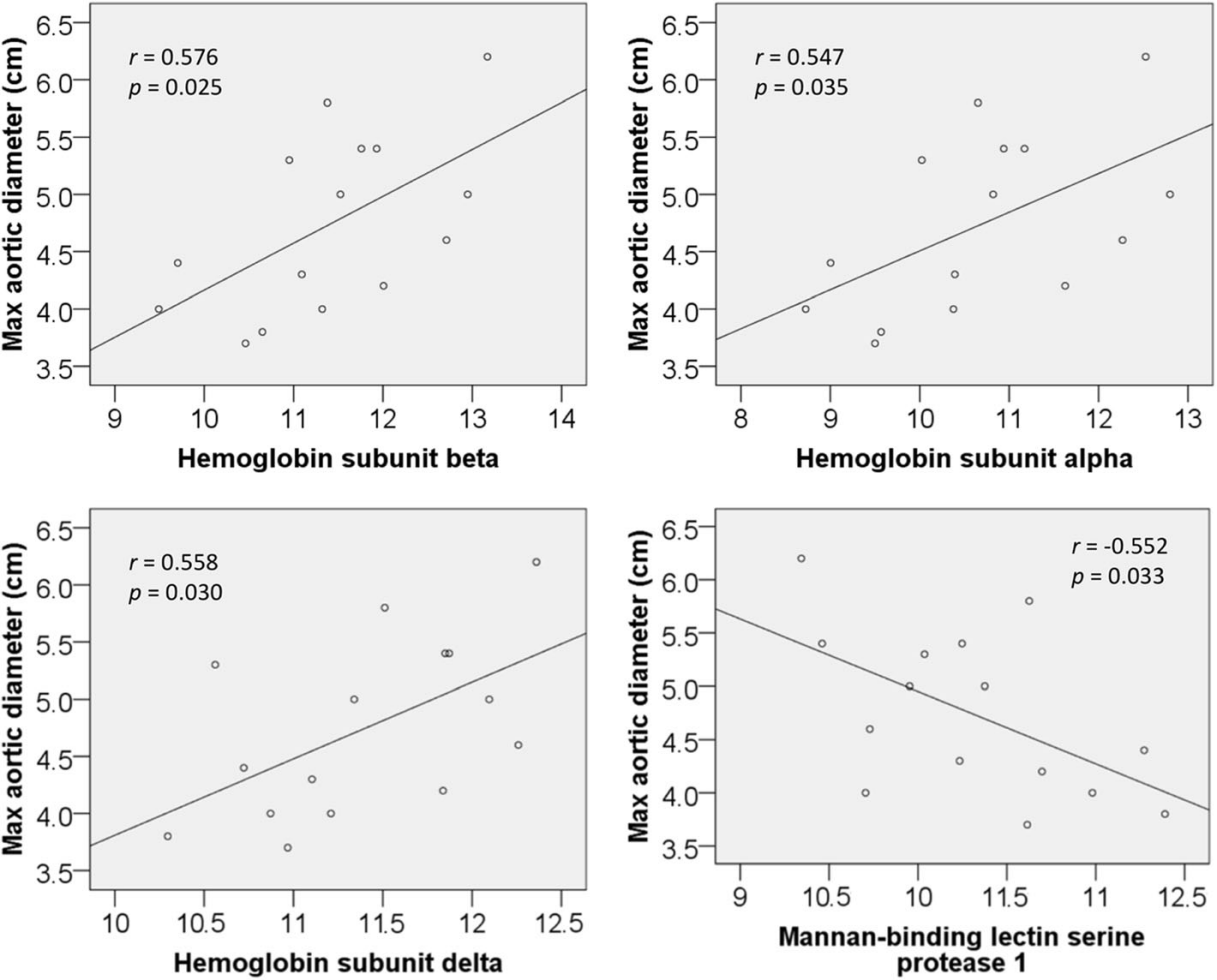
Scatter plots displaying the significant relationships between four plasma proteins and maximum aortic diameter in BAV patients (*n* = 15). Protein expressions are log2 values of SWATH-MS abundance

Linear regression analysis of plasma proteins revealed four significant (*p* < 0.05) predictors of maximum aortic diameter in BAV patients (Table 2). Haemoglobin subunits alpha, beta and delta correlated positively with increasing maximum ascending aortic diameter, and Mannan-binding lectin serine protease correlated negatively (Fig. 1). All four proteins demonstrated moderate correlation (*r* = 0.5 to 0.6). Of note, the patient’s pre-operative serum haemoglobin did not correlate significantly with the maximum aortic diameter (*p* = 0.279).

**Table 2:** Significant predictors of maximum aortic diameter in BAV patients

Plasma Protein	Pearson coefficient (*r*)	P-value	Gradient (*m*)	y-intercept (*c*)
Haemoglobin subunit beta	0.576	0.025	0.410	0.066
Haemoglobin subunit delta	0.558	0.030	0.670	−2.552
Haemoglobin subunit alpha	0.547	0.035	0.339	1.119
Mannan-binding lectin serine protease 1	−0.552	0.033	−0.679	11.745

### Plasma biomarkers to predict risk of future aortopathy

**Fig. 2:**
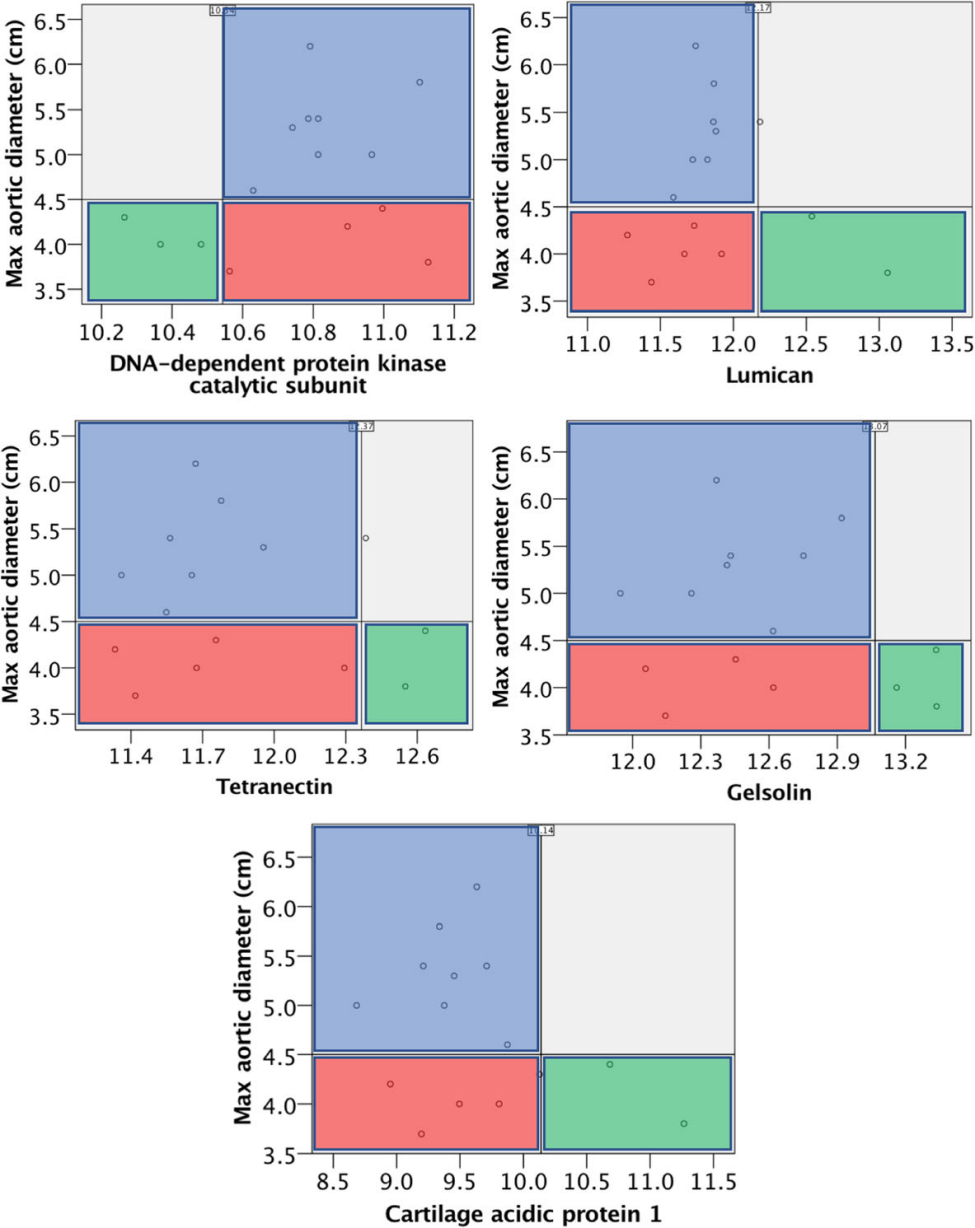
Scatter plots of candidate plasma biomarkers for predicting risk of aortopathy in non-aneurysmal BAV patients. Horizontal lines at 4.5 cm indicate the study’s division between non-aneurysmal and aneurysmal ascending aortic diameter. Vertical lines indicate mean expression in the aneurysmal group ± 2 standard deviations. Blue area = aneurysmal BAV cohort expressions upto mean ± 2 standard deviation; red area = hypothetically ‘at risk’ non-aneurysmal BAV patients; and green area = hypothetically not ‘at risk’ non-aneurysmal patients. Protein expression is given as log2 SWATH-MS abundance

In order to identify potential plasma protein biomarkers predictive of future aneurysm risk, subgroup analysis was performed on the BAV non-aneurysmal group. Five candidate plasma protein markers exhibiting low variance in the aneurysmal group but high variance in the non-aneurysmal group were identified as potential predictors of future aortopathy (Fig. 2). These were DNA-dependent protein kinase catalytic subunit, lumican, tetranectin, gelsolin and cartilage acidic protein 1.

In addition, seven proteins were identified in the plasma from all patients with aneurysmal aortas but only identified in a subset of the non-aneurysmal group. These were matrin-3, glucose-6-phosphate isomerase, coactosin-like protein, peptidyl-prolyl cis-trans isomerase A, golgin subfamily B member 1, myeloperoxidase and 2′-deoxynucleoside 5′-phosphate N-hydrolase 1. Table 3 summarises the plasma proteins implicated above.

**Table 3:** Candidate plasma biomarkers for predicting risk of aneurysm in BAV patients with non-aneurysmal aortas

Protein Name	Uniprot ID	Function	Up- or downregulated^a^
DNA-dependent protein kinase catalytic subunit	P78527	Serine/threonine-protein kinase, molecular sensor for DNA damage.	Up
Lumican	P51884	Collagen fibril organization, epithelial cell migration and tissue repair.	Down
Tetranectin	P05452	Binds plasminogen and isolated kringle 4. Packaging molecules for exocytosis.	Down
Gelsolin	P06396	Key regulator of actin filament assembly and disassembly.	Down
Cartilage acidic protein 1	Q9NQ79	Upregulated by BMP4 in mesenchymal stem cells undergoing chondrogenic differentiation.	Down
Matrin-3	P43243	Transcription and interaction with other nuclear matrix proteins to form the internal fibrogranular network.	N/A
Glucose-6-phosphate isomerase	P06744	Glycolytic enzyme and angiogenic factor (AMF) that stimulates endothelial cell motility.	N/A
Coactosin-like protein	Q14019	Binds to F-actin in a calcium-independent manner.	N/A
Peptidyl-prolyl cis-trans isomerase A	P62937	Accelerates the folding of proteins.	N/A
Myeloperoxidase	P05164	Produces hypochlorous acid (HOCl) used by the neutrophils to kill bacteria and other pathogens.	N/A
Golgin subfamily B member 1	Q14789	Forms intercisternal cross-bridges of the Golgi complex.	N/A
2′-deoxynucleoside 5′-phosphate N-hydrolase 1	O43598	Catalyses cleavage of the N-glycosidic bond of deoxyribonucleoside 5′-monophosphates.	N/A

Biomarker name, Uniprot ID, function and direction of change are given

^a^Direction of change in plasma biomarker that indicates a patient may be at risk of future aortopathy. ‘N/A’ (not applicable) indicates that presence of the protein in plasma alone may indicate risk of future aortopathy

## Discussion

The two main findings of this study are that four plasma proteins were identified as potential biomarkers for monitoring maximum aortic diameter in BAV patients, and 12 plasma proteins were identified as potential indicators for future aneurysm risk in BAV patients with non-aneurysmal aortas.

The advantage of SWATH-MS is that all proteins within a plasma sample can be detected and quantified, raising the possibility of discovering previously unknown relationships between these biomarkers and ascending aortic diameter in BAV patients. This study identified four unique plasma biomarkers that correlated significantly with maximum ascending aortic diameter in BAV patients. Previous studies have identified plasma biomarkers that correlate with aortic dimension in BAV disease. Tzemos et al. found MMP-2 levels correlated significantly with ascending aortic diameter, findings that are supported by the more recent work of Wang et al. [11, 14]. However, Abaci et al. found no correlation between MMP-2 and ascending aortic diameter [20]. In the present study, MMP-9 and 14 were elevated in the order of 11 and 41% respectively in the aneurysmal group but this was not statistically significant (*p* = 0.767 and *p* = 0.242 respectively). Drapisz et al. found a positive correlation between ADMA (eNOS inhibitor) and aortic annulus diameter (*r* = 0.4, *p* = 0.043) and Kilickesmez et al. demonstrated a significantly negative correlation between ascending aortic diameter and α1-antitrypsin level (*r* = − 0.300, *p* = 0.006) [12, 13].

In this study, haemoglobin subunits alpha, beta and delta were found to correlate significantly with ascending aortic diameter in BAV patients. In adults, two alpha and two beta subunits combine to form one haemoglobin A molecule (HbA), which comprises approximately 97% of circulating haemoglobin. Combination of two alpha and two delta subunits forms haemoglobin A2, which together with fetal haemoglobin (HbF) makes up the remaining 3% of circulating haemoglobin [21]. Supporting these findings pre-operative serum haemoglobin (Hb) was significantly increased in the aneurysmal group versus non-aneurysmal but did not correlate significantly with maximum aortic diameter. This is a novel observation as to the best of our knowledge, there are no previous reports of this association in the literature. In abdominal aortic aneurysms (AAA) however, trapping of erythrocytes within areas of intraluminal thrombus may lead to haemolysis and subsequent release of Hb, heme and iron, which liberates reactive oxygen species (ROS) [22]. Erythrocytes and heme deposits are also phagocytosed by immune cells in the aortic wall [23]. Nevertheless, intraluminal thrombus is not commonly associated with BAV ascending aortic aneurysms and inflammation is classically absent [24, 25]. The lack of any previous reports of these observations requires further investigation and validation in a larger patient cohort.

A major goal in the treatment of BAV aortopathy is developing a method of predicting which patients will develop aneurysms. To date, only one study has attempted to do this by subdividing BAV patients with non-aneurysmal aortas based on risk of future aortopathy but using aortic tissue samples instead of blood biomarkers. Grewal et al. quantified an array of cellular markers in ascending aortic tissue samples from aneurysmal and non-aneurysmal BAV and TAV patients [26]. TGF-β and pSmad2 (markers of vascular remodelling) were significantly reduced in a proportion of the non-aneurysmal BAV group versus the other non-aneurysmal patients and the aneurysmal BAV group. The study concluded that a subgroup of BAV patients with non-aneurysmal aortas appear to have more active remodelling than others, which could represent susceptibility to developing aneurysm. To our knowledge, the paper by Grewal et al. is the first to attempt to subdivide non-aneurysmal BAV patients on the basis of susceptibility to developing aneurysm using biomarkers. Whilst it did not employ a plasma biomarker, which would be more practical in clinical practice, it set precedence for the future direction in detecting and preventing BAV aortopathy in susceptible individuals.

**Fig. 3:**
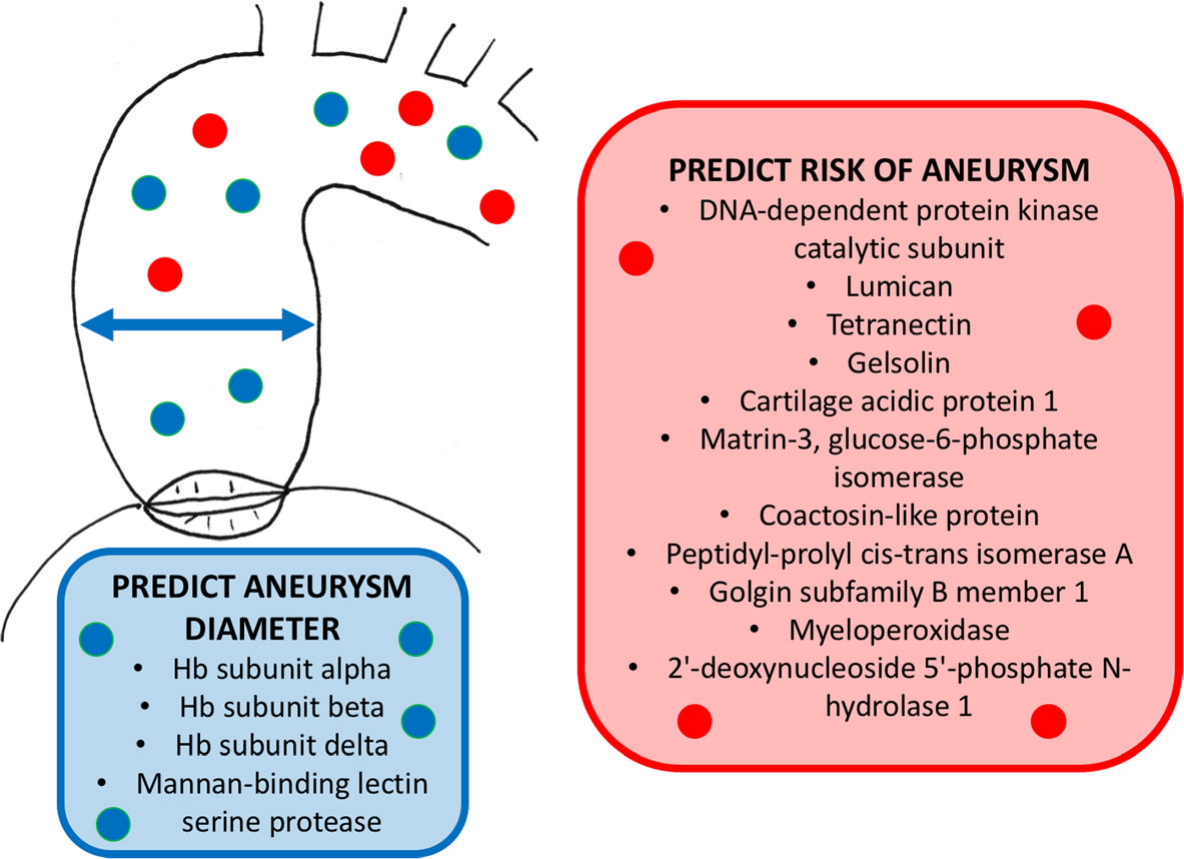
Summary diagram of the key findings in this study. Red dots represent plasma biomarkers which may be associated with an increased risk of aneurysm formation (red box). Blue dots represent plasma biomarkers which predict aneurysm diameter (blue box)

The present study is the first to utilise SWATH-MS to quantify all detectable proteins in blood plasma samples and identify potential biomarkers which may predict ‘at risk’ non-aneurysmal BAV patients. The principle advantage of blood over a tissue biomarker is that plasma samples are more easily obtainable, in comparison to ascending aortic tissue, which is less practical. Therefore, a result can be obtained from a blood test and a treatment plan implemented long before aneurysm develops. Twelve potential plasma biomarkers were identified. Of note, the ‘at risk’ patients demonstrated increased DNA-dependent protein kinase catalytic subunit, a protein associated with DNA damage, which may reflect the increased remodelling and vascular smooth muscle cell (VSMC) apoptosis occurring in BAV aortopathy. Similarly, down regulation of two key extracellular matrix (ECM) and VSMC cytoskeletal assembly proteins lumican and gelsolin were also observed in the ‘at risk’ profile. This is concurrent with accelerated ECM degradation and VSMC loss observed in BAV aortopathy [27, 28]. Therefore, these biomarkers may reflect underlying pathological processes occurring in the ascending aorta and could represent entities with which to predict future aortopathy in BAV patients. A summary diagram of the key findings of this study can be found in Fig. 3.

A major limitation of this pilot study was not validating candidate biomarkers through follow-up of changes in ascending aortic diameter of the non-aneurysmal patients over time. This was not possible within the time scale of this study but should be included in the design of a future prospective validation investigation. Furthermore, in concluding that our candidate biomarkers may predict future aneurysm based on similar expression to aneurysmal patients, our assumption was that expression of the biomarkers remains constant. However, we cannot exclude the possibility that expression may vary over time and a validation study is required to confirm expression stability. Another limitation of this study was the small sample size. The main reason for this is the time taken to perform detailed informatics analysis of the data generated is considerable, therefore large sample numbers were not feasible within the time scale of this study. With small sample numbers, we cannot exclude the possibility that differences in the patient’s demographics (e.g. statin use and valve morphology) may have influenced the differences in protein expression described in this study. Furthermore, we were unable to correlate the plasma findings in paired tissue samples, although this is a subject of ongoing investigation. In addition, TAV control groups would have proved useful to ensure that the proteins identified were indeed unique to BAV aortopathy. However, investigation of TAV aneurysms was not the focus of this study, and the biomarkers identified may still differentiate ‘at risk’ BAV patients regardless of their presence in TAV patients. Finally, this study did not use correction for multiple testing, despite identifying a large number of candidate biomarkers. Whilst this has the potential to introduce falsely positive biomarkers, the priority of this study was to identify of all potential biomarkers for future investigation, rather than excluding those with lower potential value.

## Conclusions

This study identified a number of candidate predictors for aortic diameter and future risk of aneurysm in BAV patients. It provides valuable data and proof of principle that could be used to design a large scale prospective investigation of several hundred BAV patients. Plasma levels of candidate biomarkers need to be quantified in a large population of BAV patients and matched with changes in ascending aortic diameter from serial follow-up imaging, allowing direct correlation with disease progression. Ultimately, a more affordable ‘off-the-shelf’ follow-on blood assay could then be developed in place of SWATH-MS for use in the healthcare setting.

## Additional file

Additional file 1: Supplementary methods. (DOCX 25 kb)
