# Supplementary material for: Candidate plasma biomarkers for predicting ascending aortic aneurysm in bicuspid aortic valve disease
Source: J Cardiothorac Surg. 2018 Jun 22;13:76. doi: 10.1186/s13019-018-0762-1 (PMC6013884; doi:10.1186/s13019-018-0762-1)
Supplement: Supplementary file 1 — Supplementary methods. (DOCX 25 kb) [file 13019_2018_762_MOESM1_ESM.docx]

# **Candidate plasma biomarkers for predicting ascending aortic aneurysm in bicuspid aortic valve disease**

**Journal of Cardiothoracic Surgery**

*Oliver J. Harrison – ojharrison@doctors.org.uk^1,2^

Felino Cagampang – F.Cagampang@soton.ac.uk^1^

Sunil K. Ohri – Sunil.Ohri@uhs.nhs.uk^2^

Christopher Torrens – c.torrens@soton.ac.uk^1^

Kareem Salhiyyah – Kareem.Salhiyyah@uhs.nhs.uk^2^

Amit Modi - drmodiamit@hotmail.com^3^

Narain Moorjani – narainmoorjani@hotmail.com^4^

Anthony D. Whetton – tony.whetton@manchester.ac.uk^5, 6^

Paul A. Townsend – paul.townsend@manchester.ac.uk^5, 6^

^1^Institute of Developmental Sciences, Faculty of Medicine, University of Southampton, Southampton, United Kingdom

^2^Department of Cardiac Surgery, University Hospital Southampton, Southampton, United Kingdom

^3^Sussex Cardiac Centre, Brighton, United Kingdom

^4^Department of Cardiac Surgery, Papworth Hospital NHS Foundation Trust, University of Cambridge, United Kingdom

^5^Stoller Biomarker Discovery Centre, Manchester Academic Health Science Centre, University of Manchester, Manchester, United Kingdom

^6^Division of Cancer Sciences, Faculty of Biology, Medicine and Health, Manchester Cancer Research Centre, Manchester Academic Health Science Centre, University of Manchester, Manchester, United Kingdom

**Corresponding author:**

Mr Oliver Harrison

Department of Cardiac Surgery

D-level, North Wing (MP 46)

Southampton General Hospital

Tremona Road

Southampton

SO16 6YD

### SWATH-MS quality control

Plasma samples were subject to SWATH-MS to identify and quantify the abundance of plasma proteins (performed at the MRC-funded Stoller Biomarker Discovery Centre, University of Manchester, UK)[1]). To ensure accuracy of results each sample was normalised to its own protein concentration before processing. In addition, index ‘Retention Time’ peptides at known concentrations were added to each sample for the MS runs, acting as an internal standardisation control. A control human plasma sample (Seralab, UK) sourced from a healthy male donor was run in parallel to the experimental samples and subject to gel electrophoresis to check protein digest efficiency prior to SWATH-MS. Finally, as per our SOP, the Stoller Centre ran twice daily quality control checks using a standard K562 cell line protein digest to continually monitor instrument performance throughout the sample batch.

### Plasma sample pre-processing (immunodepletion)

Plasma was centrifuged at 4^o^ C (15,700 g for 5 min) to remove particulates. Major plasma proteins were removed by immunodepletion using Pierce Top 12 abundant protein depletion spin columns (Thermo Fisher Scientific, UK). Plasma samples were then concentrated and buffer exchanged to 50 mM Ammonium Bicarbonate using Amicon Ultra 0.5 ml 3K Centrifugal filters (Merck Millipore, USA) and the protein concentration measured using the Pierce BCA Protein Assay according to manufacturer’s instructions (Thermo-Fisher Scientific, UK).

### Reduction, alkylation and digestion of non-depleted serum

Depleted plasma (40 μg) was aliquoted and normalised to 90 µL with LCMS water (VWR chemicals, UK). A 20 µL aliquot of tris-(2-carboxyethyl)-phosphine hydrochlorine (TCEP, 66 mM – in 0.6 M TEAB; Sigma Aldrich, UK) was added to each sample and these were incubated at 60° C in a shaking heater block (VWR) for 1 hour at 300 rpm. Following incubation, a 10 µL aliquot of iodoacetamide (72 mM – in 0.1 M TEAB; Sigma Aldrich, UK) was added to each sample. These were covered in aluminium foil and placed into the dark for 30 minutes. During the incubation, trypsin (sequencing grade modified trypsin in 0.1 M TEAB; Promega, UK) was prepared in a ratio 1:20 trypsin-protein for each sample. Samples were removed from the dark and 10 µL of 0.1µg/µL trypsin was added to each. Digestions were incubated at 37° C for 3 hours at 300 rpm. Samples were then acidified with 15 µL of formic acid (10%).

### Data acquisition

Samples were placed into a miVac vacuum centrifuge (Genevac) in a water programme without heat and were dried overnight. A master mix of Biognosys iRT + SCIEX pepcalmix + loading buffer (2% acetonitirile, 0.1% formic acid; Sigma-Aldrich, UK) was prepared and a 72 µL aliquot was added to each dried sample and vortexed. The samples were transferred to HPLC vials and placed into the HPLC eksigent 425 to run in microflow (10 µg/injection) through the SCIEX TripleTOF 6600 variable window mass spectrometer with a 120-minute run time.

### Data analysis

Protein identification and quantification was performed using the 10,000 protein spectral library described in Rosenberger *et al.* and targeted data acquisition based on the extraction of fragment ion chromatograms for each peptide of interest from SWATH-MS maps[2]. The maps were analysed for protein IDs using an open source data analysis pipeline, including OpenSWATH, PyProphet and TRIC Feature Aligner, as described in previous publications[3-5]. These algorithms detect peaks (fragment ion signatures for the peptides) and assigns a quantitative value to them. First, peptides are identified from their chromatogram (extracted-ion chromatogram; XIC) signature. A start and finish of each peak (creating a curve) is then defined and finally the area under the curve is calculated. The algorithm returns a peptide abundance value equivalent to the sum of areas for the peaks (known as peak groups). The area under the curve is equivalent to the peptides relative abundance. Abundance values were then entered into a MS-proteomics quantitative package MSstats[6] and subjected to the following:

1. Overall peak group intensity value split into the individual peak intensity (quantification relies the individual fragments).
2. The software applies normalisation methods (median correction) so that samples are comparable to each other (correcting for errors resulting from machine or sample preparation variations).
3. Following normalisation, MSstats takes individual fragment intensities and applies a statistical model to estimate the abundance of the protein the fragment maps on to.

Calculated protein ‘abundance’ is an estimated value (based on the intensity values of the fragment ions) that has been corrected for error. Values are presented as log2 abundance.

**References**

1. **Stoller Biomarker Discovery Centre** [<http://www.biomarkers.manchester.ac.uk/about/sbdc/>]

2. Rosenberger G, Koh CC, Guo T, Röst HL, Kouvonen P, Collins BC, Heusel M, Liu Y, Caron E, Vichalkovski A *et al*: **A repository of assays to quantify 10,000 human proteins by SWATH-MS**. *Scientific Data* 2014, **1**:140031.

3. Teleman J, Rost HL, Rosenberger G, Schmitt U, Malmstrom L, Malmstrom J, Levander F: **DIANA--algorithmic improvements for analysis of data-independent acquisition MS data**. *Bioinformatics (Oxford, England)* 2015, **31**(4):555-562.

4. Rost HL, Liu Y, D'Agostino G, Zanella M, Navarro P, Rosenberger G, Collins BC, Gillet L, Testa G, Malmstrom L *et al*: **TRIC: an automated alignment strategy for reproducible protein quantification in targeted proteomics**. *Nature methods* 2016, **13**(9):777-783.

5. Rost HL, Rosenberger G, Navarro P, Gillet L, Miladinovic SM, Schubert OT, Wolski W, Collins BC, Malmstrom J, Malmstrom L *et al*: **OpenSWATH enables automated, targeted analysis of data-independent acquisition MS data**. *Nature biotechnology* 2014, **32**(3):219-223.

6. **Statistical Tool For Quantitative Mass Spectrometry-Based Proteomics** [<http://msstats.org/>]
